# Spectral Studies of UV and Solar Photocatalytic Degradation of AZO Dye and Textile Dye Effluents Using Green Synthesized Silver Nanoparticles

**DOI:** 10.1155/2016/8629178

**Published:** 2016-06-13

**Authors:** R. Mariselvam, A. J. A. Ranjitsingh, P. Mosae Selvakumar, Abdullah A. Alarfaj, Murugan A. Munusamy

**Affiliations:** ^1^Department of Chemistry, Sri Paramakalyani College, Alwarkurichi, Tirunelveli, Tamil Nadu 627412, India; ^2^J.P. College of Arts and Science, Ayikudi, Tamil Nadu 627852, India; ^3^Department of Chemistry, Karunya University, Coimbatore, Tamil Nadu 641114, India; ^4^Department of Botany and Microbiology, College of Science, King Saud University, Riyadh 11451, Saudi Arabia

## Abstract

The photocatalytic degradation of the chemical dye AZO and dye effluents in different time duration has been investigated using biologically synthesized silver nanoparticles. Dye industry effluents and AZO dye undergo degradation to form harmless intermediate and colourless products following irradiation by UV and solar light in the presence of green synthesized silver nanoparticles. The degree of degradation was tested under the experimental conditions such as P^H^, temperature, and absorbance of the dye in UV and solar light was measured. The degradation was higher in the UV light source than in the solar light source. Green synthesized silver nanoparticles in the UV light source were found to expedite the dye degradation process.

## 1. Introduction

The emission of effluents from textile industries has been a major concern of the modern world, due to the great pollution that these effluents promote on the water resources [1]. In the synthetic dyes released in effluents from textile industries, AZO dye is one of the more detrimental classes because of its high persistent nature in the aquatic environment, due to its chemical compositions, involving aromatic rings, azoic linkages, and amino groups [2]. The dye industry effluents are a major problem worldwide. The dye industry effluents not only disturb the aquatic ecosystem but also the biota and human beings.

The dyes are used to modify the colour characteristics of different substrates, such as fibre products, fabric, leather, plastic, wood products, and paper. The dyes were extracted from natural sources, mainly from animals and plant materials, and used much before the nineteenth century [3]. After the nineteenth century the natural dyes were replaced by synthetic dyes. Every day new synthetic dyes are developed for new colours and this eliminates the use of natural dye. These synthetic dyes are inorganic substances to pollute the water bodies. The dye effluent can induce oxidative stress [4], mutagenic activity [5–8], and carcinogenic activity and it can also generate DNA adducts and so forth in living organisms. Nowadays, the dyestuffs and textile effluents were treated using various physical, chemical, and biological methods [9–14]. In the dye effluents treatment, the use of green synthesized nanoparticles is less understood. So in the present study silver nanoparticles synthesized using plant material were tested to treat dye effluents.

## 2. Materials and Methods

### 2.1. Synthesis and Characterization of Silver Nanoparticles (AgNPs)

The green synthesized silver nanoparticles were prepared using the crude extract of coconut tree inflorescence fraction (EA : M (40 : 60)) as per technology developed [15]. These synthesized nanoparticles were 22 nm, spherical sized particles. The characteristics of AgNPs were studied using UV/visible spectroscopy, FTIR, and TEM assays.

### 2.2. Preparation of AZO Dye

The commercially available AZO dye was purchased from Merck, India. One gram of AZO dye was dissolved in 1000 mL of double distilled water carefully in dark bottles. The dye concentration was 0.1%. The prepared dye was used for further studies.

### 2.3. Collection of Dye Industry Effluents

The textile mill effluent was collected from the sewage tank in a textile factory in Tiruppur, Tamil Nadu, India.

### 2.4. Photocatalytic Based Degradation

The photocatalytic degradation of AZO dye and textile industry effluents was investigated using green synthesized silver nanoparticles as a photocatalyst under solar light and UV light. The dye degradation was tested by UV/visible double beam spectroscopy 2203 and kinetic measurement was performed at room temperature for UV radiated dye degradation and at outside room temperature for solar radiation. The concentration of the dye was (Optical Density) measured at 200–1000 nm wavelength. The progress of the photocatalytic reaction was observed by recording optical density at regular time intervals. The temperature and P^H^ were also measured at regular time intervals.

## 3. Result and Discussion

AZO dyes are compounds characterized by the presence of one or more AZO groups (-N=N-) [2]. This was confirmed by the presence of two absorption bands at 568–737 nm and another at 404–475 nm. These two absorption bands are responsible for *n* → *π* ^*∗*^ electron transition. The effluents have two major absorption peaks and were noted at 227 nm and 214 nm. These two peak values always correspond with *n* → *π* ^*∗*^ electron transition.

### 3.1. Photodegradation Process for AZO Dye

**Figure 1:**
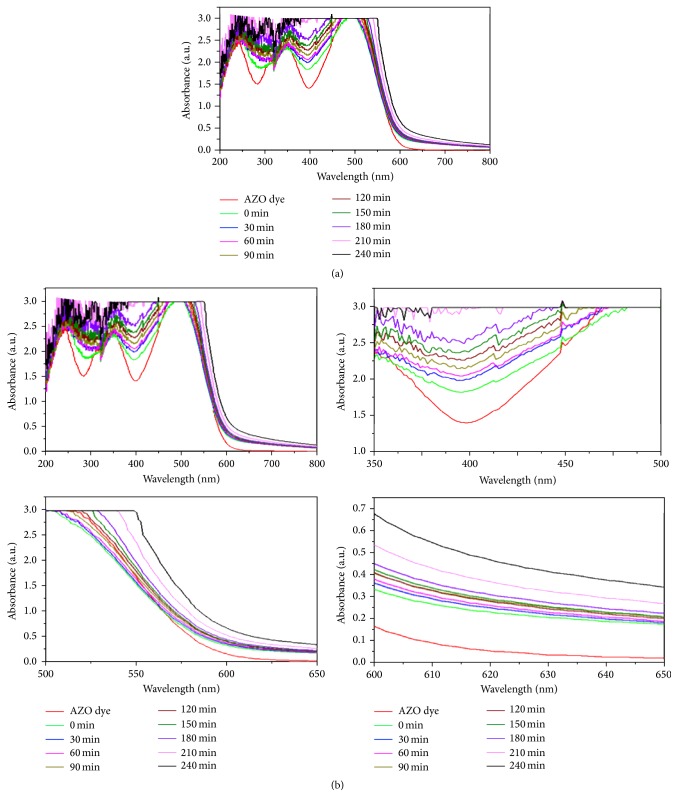
Solar light irradiated AZO dye degradation using green synthesized AgNPs as photocatalyst.

The photodegradation process was carried out under solar and UV light irradiation using green synthesized silver nanoparticles as photocatalysts. The absorption spectra of AZO dye (Figures 1(a) and 1(b)) degradation under solar light irradiation were observed regularly at time intervals. The solar and UV light irradiation, the intimacy between dye molecules and a semiconductor material, and the presence of oxygen are indispensable for an efficient photosensitized degradation reaction.

**Figure 2:**
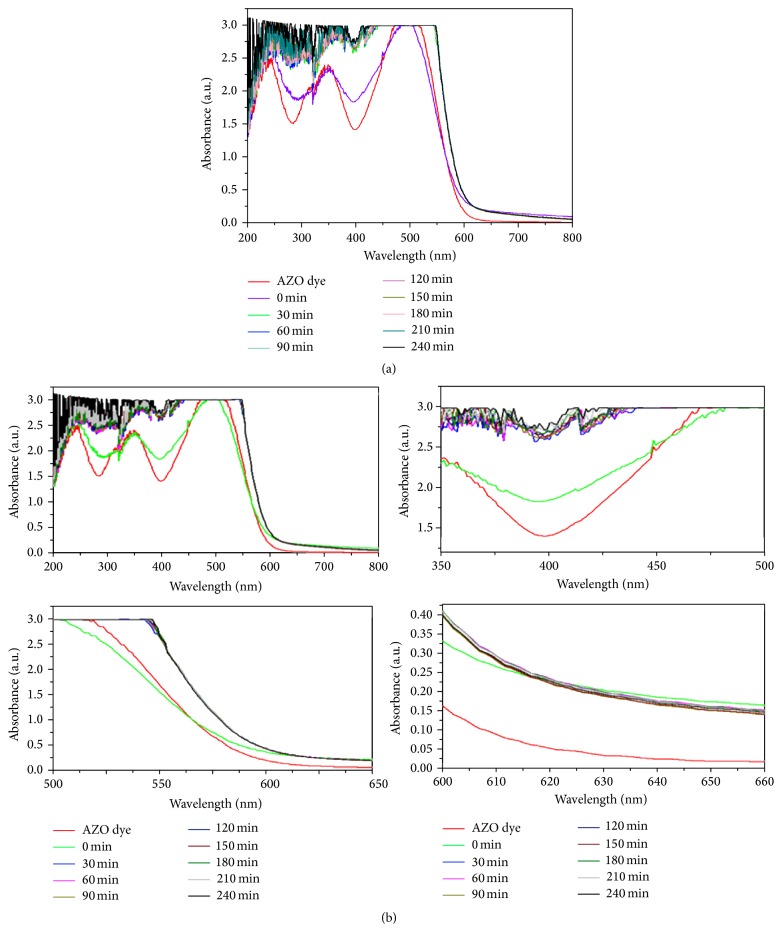
Monochromatic UV light irradiated AZO dye degradation using green synthesized AgNPs as the photocatalyst.

**Figure 3:**
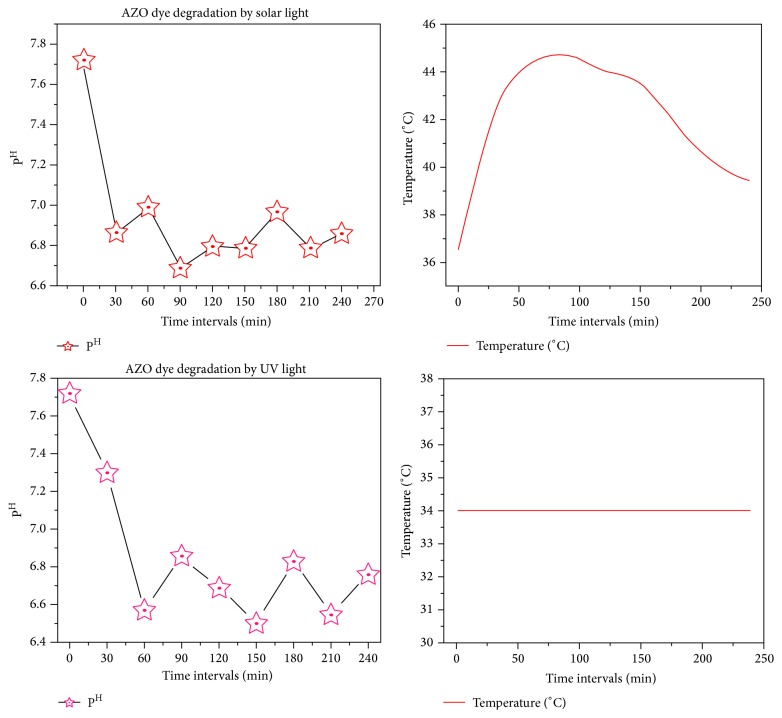
P^H^ and temperature variation ranges in different time intervals of AZO dye degradation.

The results were shown in Figures 1(a), 1(b), 2(a), and 2(b). The excitation of AZO molecules also depends on the P^H^ and temperature. The excited molecules were absorbed by the Ag nanoparticles. It was confirmed by the absorption spectrum at the regular time intervals (Figures 1(b) and 2(b)). The time taken for dye degradation and P^H^ changed irregularly (Figure 3). The efficacy of light source has changed the electron transition in AZO molecules.

As the dye molecules were largely seen on the sample surface, the degradation reaction proceeds predominantly through the monochromatic UV light irradiation as reflected by the drastic shift in absorption spectra as shown in Figures 2(a) and 2(b). The comparison of solar and UV light irradiation showed that the UV light has degraded the AZO dyes more effectively than the solar sources.

### 3.2. Photodegradation Process for Textile Industry Effluents

The photodegradation process was carried out under solar and ultraviolet irradiation using green synthesized silver nanoparticles as the photocatalyst. Figures 5(a) and 5(b) indicate the absorption spectra of effluent degradation under solar light irradiation. The solar light irradiation induced the electrons (nanoparticles) and is excited from *n* → *π* ^*∗*^ electron transition. The absorption rate at regular time intervals and the P^H^ sequentially increased (Figure 6) based on the light intensity (light energy).

In this reaction also the solar and UV light irradiation, the intimacy between the dye molecule's and a semiconductor material, and the presence of oxygen are indispensable for an efficient photosensitized degradation reaction.

**Figure 4:**
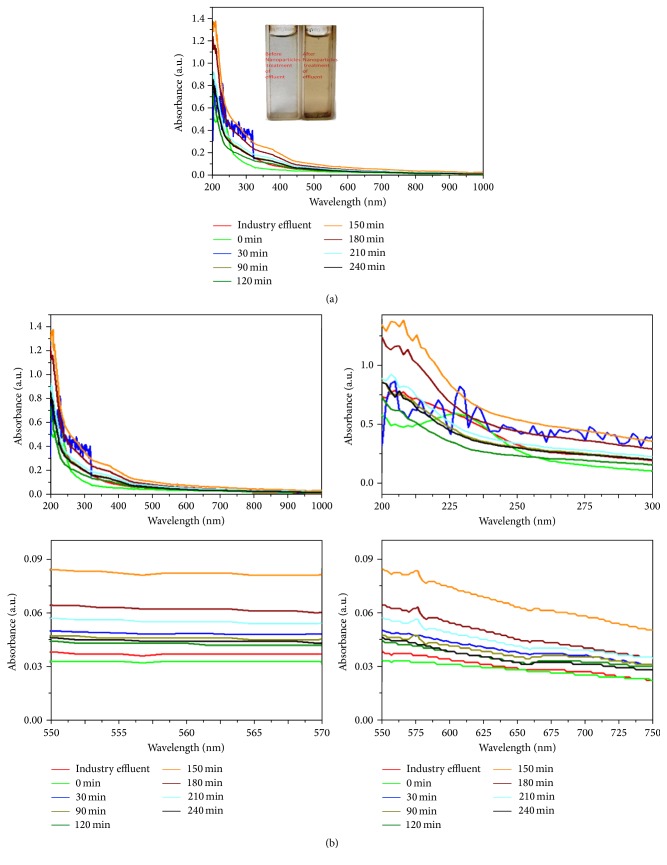
Solar light irradiated industry effluents using green synthesized AgNPs as the photocatalyst.

**Figure 5:**
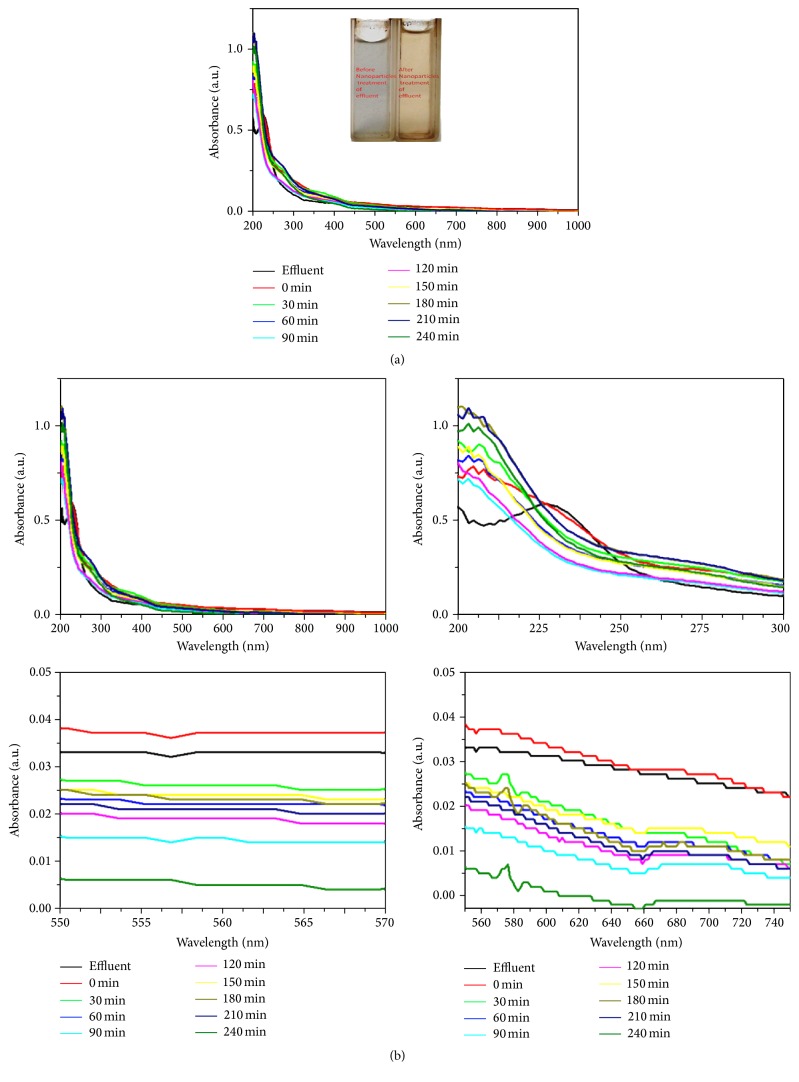
Monochromatic UV light irradiated industry effluent using green synthesized AgNPs as the photocatalyst.

**Figure 6:**
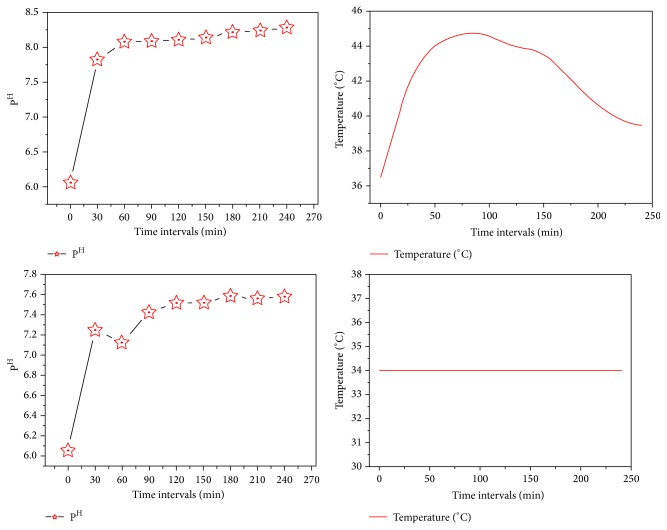
P^H^ and temperature variation ranges in different time intervals of industry effluent degradation.

The results shown in Figures 4(a) and 5(a) show the excitation of dye molecule's in the effluents. The excitation of electrons depends on P^H^, light intensity (light energy), and temperature. The excited electrons bound to the dye molecules. Then the effluent got changed in their physical, chemical, and biological properties and binding capability. This was confirmed by the absorption spectrum at the regular time intervals (Figures 4(b) and 5(b)). P^H^ got increased with the increase of the duration of dye degradation (Figure 6).

## 4. Conclusion

Presence of AZO dyes and other dyes in the textile mill effluents threatens the aquatic food chain. In order to degrade the dyes, UV and solar light irradiation in the presence of green synthesized silver nanoparticles were tested. The presence of green synthesized AgNPs promoted the degradation fast. Hence the coconut tree inflorescence extract mediated silver nanoparticles were found to enhance the photocatalytic dye degradation effectively.
